# Host heterozygosity and genotype rarity affect viral dynamics in an avian subspecies complex

**DOI:** 10.1038/s41598-017-13476-z

**Published:** 2017-10-17

**Authors:** Justin R. Eastwood, Raoul F. H. Ribot, Lee Ann Rollins, Katherine L. Buchanan, Ken Walder, Andrew T. D. Bennett, Mathew L. Berg

**Affiliations:** 1 Centre for Integrative Ecology, School of Life and Environmental Sciences, Deakin University, Geelong, 3216 Victoria, Australia; 2 School of Biological Sciences, Monash University, Melbourne, Victoria, 3800 Australia; 3 Metabolic Research Unit, School of Medicine, Deakin University, Geelong, 3216 Victoria, Australia

## Abstract

Genetic diversity at community, population and individual levels is thought to influence the spread of infectious disease. At the individual level, inbreeding and heterozygosity are associated with increased risk of infection and disease severity. Host genotype rarity may also reduce infection risk if pathogens are co-adapted to common or local hosts, but to date, no studies have investigated the relative importance of genotype rarity and heterozygosity for infection in a wild, sexually reproducing vertebrate. With beak and feather disease virus (BFDV) infection in a wild parrot (*Platycercus elegans*), we show that both heterozygosity and genotype rarity of individual hosts predicted infection, but in contrasting ways. Heterozygosity was negatively associated with probability of infection, but not with infection load. In contrast, increased host genotype rarity was associated with lower viral load in infected individuals, but did not predict infection probability. These effects were largely consistent across subspecies, but were not evident at the population level. Subspecies and age were also strongly associated with infection. Our study provides novel insights into infection dynamics by quantifying rarity and diversity simultaneously. We elucidate roles that host genetic diversity can play in infection dynamics, with implications for understanding population divergence, intraspecific diversity and conservation.

## Introduction

Pathogens by definition have negative health consequences for their host and often reduce host likelihood of survival or reproduction^1^. Therefore, pathogens have long been considered a strong selective force against susceptible host individuals^2–4^. Genetic diversity is hypothesised to influence the susceptibility of hosts to infection directly, and it is generally accepted that lower genetic diversity increases susceptibility^1^. In natural populations, individuals that are more homozygous tend to exhibit higher infection probabilities^5–9^ and pathogen loads^7,8,10,11^. A possible mechanism explaining this phenomenon could relate to the reduced capability of individuals with reduced allelic diversity at loci associated with immunity (e.g. major histocompatibility complex or Toll-like receptors) to defend themselves against pathogens^12–14^, or indirectly through negative effects associated with inbreeding (e.g. expression of deleterious recessive alleles)^15,16^. In addition, individual susceptibility may also be related to the co-adaptation of the pathogen with common genotypes, which gives rise to a selective advantage for rare genotypes^17–19^. Whilst selection against homozygotes results in greater overall population heterozygosity^2^, rare genotype advantage due to selection against common genotypes, results in negative frequency-dependent selection on genotypes^20,21^. Hence pathogens have considerable potential to contribute to the maintenance of genetic diversity and subsequently hinder or promote host divergence between wild populations^22,23^. Although studies have separately investigated roles of heterozygote advantage and negative-frequency dependent selection in pathogen susceptibility, studies are currently lacking which assess these aspects concurrently in the same system. Furthermore, while there is evidence for pathogen selection against common host species at a community level^24^ (involving common alleles involved in immunity^12,13,25^ and common clonal variants^17–19^), no study has investigated the effect of multilocus genotype rarity in a wild, sexually reproducing vertebrate. The use of highly variable species, in species complexes, such as the crimson rosella (*Platycercus elegans*), offers a unique opportunity to investigate host-pathogen interactions and determine how genetic diversity influences pathogen infection^22^.

**Figure 1:**
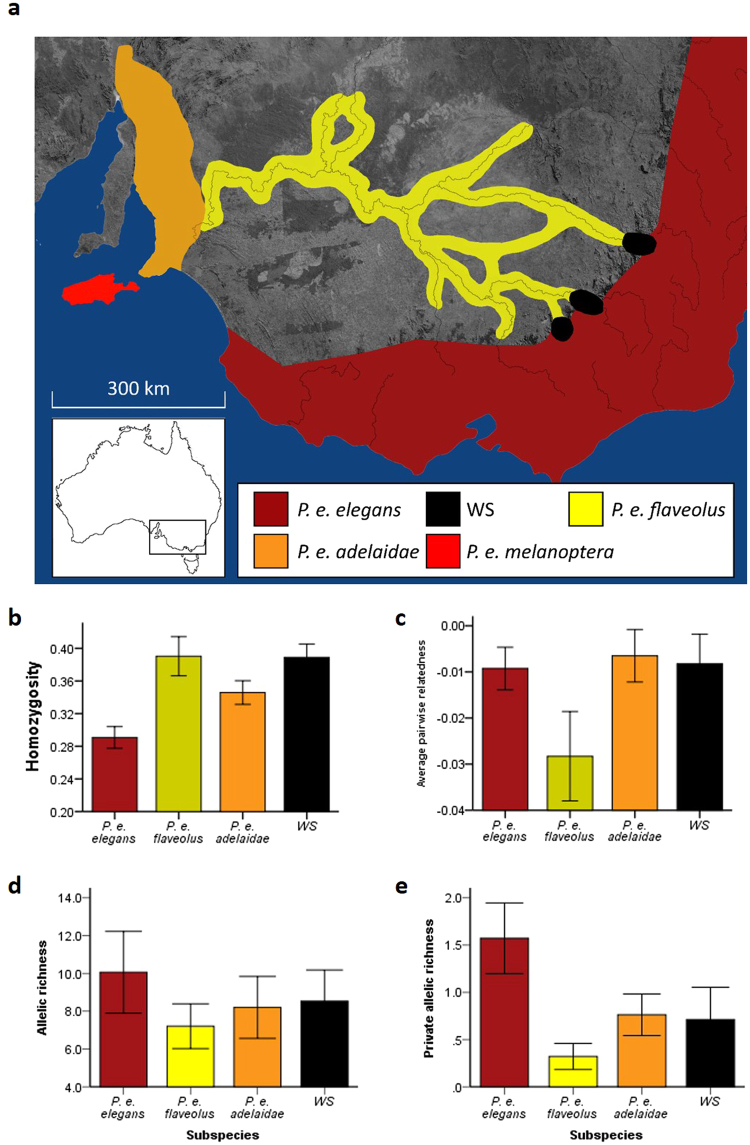
**(a)** The distribution of *Platycercus elegans* in south eastern Australia, and differences between the different subspecies and putative hybrid populations (labelled western slopes, WS) in genetic diversity parameters including **(b)** homozygosity-by-loci, **(c)** average pairwise relatedness, **(d)** allelic richness, and **(e)** private allelic richness. Error bars represent standard error of the mean. This study included samples from the Western Slopes populations and three subspecies, but not *P. e. melanoptera*. Map was modified from Eastwood *et al*.^22^.

In wild *P. elegans*, we tested whether genetic diversity influences susceptibility to a highly prevalent pathogen (beak and feather disease virus; BFDV). *P. elegans* is a common parrot which occupies diverse habitats in south eastern Australia^26^. Its distribution encircles unsuitable habitat and includes several subspecies and putative hybrid populations based primarily on plumage colouration (Fig. 1a)^27,28^. BFDV is a single-stranded DNA circovirus which consists of two primary open reading frames (ORF), the capsid (CAP), and the replication-associated ORF (REP)^29^. Associated signs of disease vary between species, but typically include feather malformation and/or loss, beak and claw deformity and immune suppression^29,30^. The latter often results in death, thereby explaining the high mortality rates and the status of BFDV as a global conservation concern^31–33^. Recently, we found that the normally highly pathogenic BFDV varied in both prevalence and infection load between the *P. elegans* subspecies^22^. These findings are consistent with the interpretation that the subspecies differ in their susceptibility to the disease and/or infection, and have implications for the evolution of population divergence in this system^22^. BFDV prevalence and load were notably lower in phenotypically intermediate forms (*P. e. adelaidae* and Western Slopes (WS)), than the two most phenotypically distinct subspecies (*P. e. elegans* and *P. e. flaveolus*)^22^; WS is a putative hybrid population where *P. e. elegans* and *P. e. flaveolus* overlap on the western slopes of the Great Dividing Range^27,28^. In addition, factors such as BFDV phylogeny, geographic location, and the susceptible host community (Psittaciforme species diversity and composition) were not associated with prevalence or load. It seems possible, therefore, that differences in individual and population level genetic diversity among these populations may explain this intraspecific variation in BFDV infection^22^.

In the study reported here, we genotyped *P. elegans* using microsatellite markers to investigate the effects of host genetic diversity on BFDV prevalence and viral load, using two distinct indices, overall genetic diversity (homozygosity-by-loci, HL) and a novel index of genotype rarity (average pairwise relatedness, APR). Our aim was to assess the roles of genome-wide heterozygosity and genotype rarity of hosts, as opposed to functional and adaptive interactions between specific host genes and the pathogen. This approach provides information on the effect of genome-wide processes on infection such as inbreeding, introgression and population dynamics^34^. To test our hypotheses that an individual’s overall heterozygosity or rarity within a population predicts infection probability or severity, we modelled whether HL or APR were associated with the likelihood of BFDV infection, or with viral load in infected hosts. Our analyses controlled for subspecies, age class and sex, which are other host traits that have previously been associated with BFDV infection^22^, and sought to confirm whether HL and APR operate independently. In addition, we investigated genetic local effects using single microsatellite loci, specifically whether pairwise host genetic distance (calculated using pairwise relatedness) was correlated with BFDV genetic distance to test if common host genotypes share similar BFDV strains, as would be predicted in an antagonistically co-evolving host-pathogen relationship^19,35^, and whether genetic diversity differed across subspecies.

## Results

### Predictors of BFDV infection status

**Figure 2:**
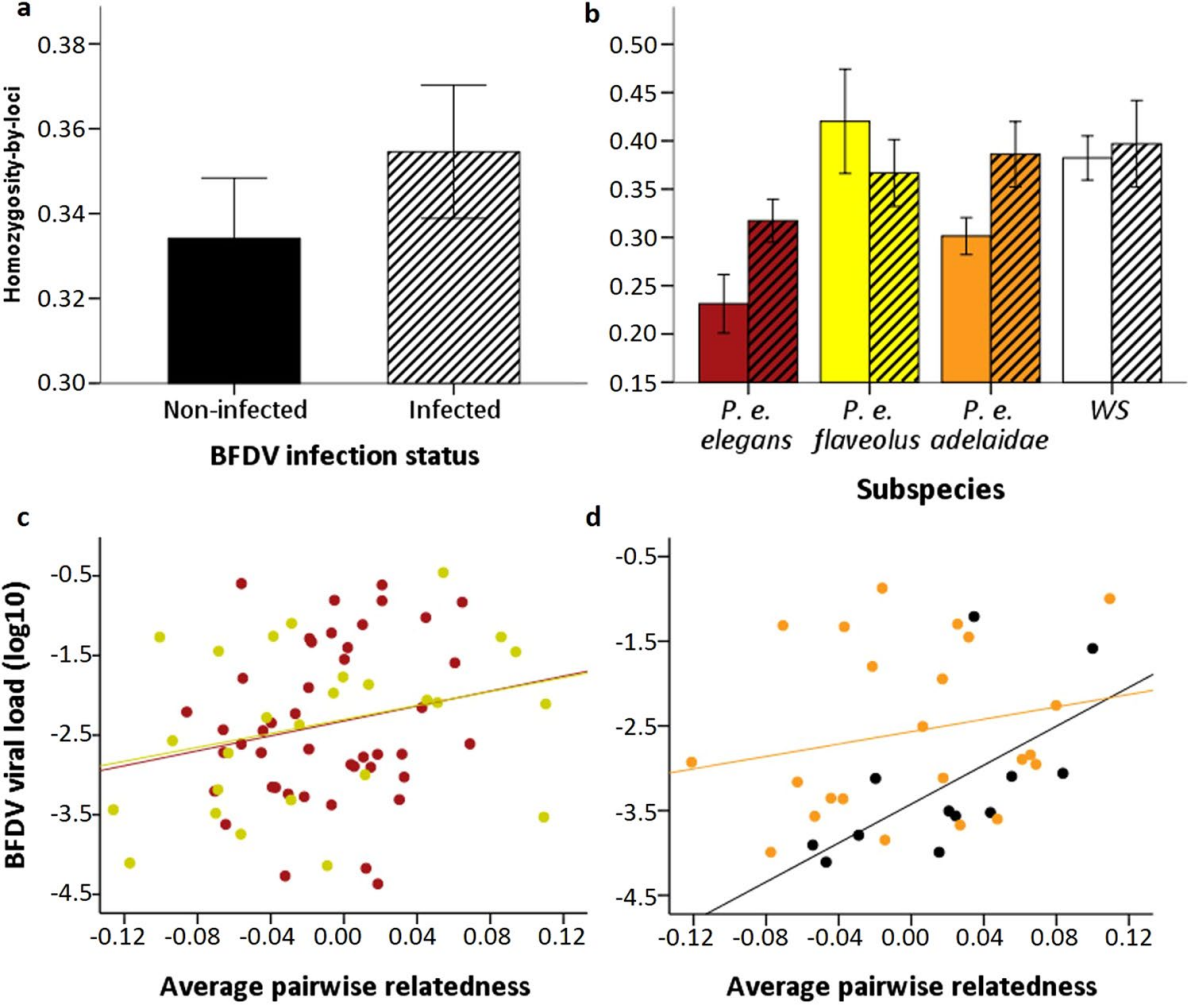
**(a)** Individual homozygosity-by-loci (HL) was higher (indicating lower heterozygosity) in BFDV infected hosts (n = 106) than non-infected hosts (n = 118); **(b)** Compared to uninfected, infected hosts had lower heterozygosity in the *Platycercus elegans elegans* (n = 53) and *P. e. adelaidae* (n = 76) subspecies, similar in the Western Slopes (WS) population (n = 65), and lower in *P. e. flaveolus* (n = 30). **(c**,**d)** Among infected hosts, viral load was positively associated with an estimate of genotype rarity (average pairwise relatedness) in all populations including *P. e. elegans* (red symbols), *P. e. flaveolus* (yellow), *P. e. adelaidae* (orange), and the WS population (black). For illustrative purposes lines of best fit for each subspecies were derived using least square regression. Interactions between subspecies and HL or APR were not significant (see methods text). We removed two outliers from (**d**) for presentation purposes but these were included in statistical analyses. Error bars in panels (**a**) and (**b**) represent ± 1 standard error.

Four plausible models were found to predict infection status, with a combined likelihood of 90.8% (Table 1). The two top ranked models had similar support (ΔAIC_c_ = 0.59) and the best model was 2.55 × 10^19^ times more likely than the null model (intercept only). All four plausible models contained the predictors subspecies and age class. HL (homozygosity-by-loci) was present in the two most highly ranked models, indicating that it was also an important predictor of infection status (Table 1); parameter weights indicated that HL was the third most important predictor after subspecies and age (Table 2). Greater HL was associated with higher infection probability with an odds-ratio of 10.79 (confidence interval 1.11–105.21; Table 2); this equates to a 26.8% (confidence interval 1.0–59.3%) greater likelihood of infection for each 0.1 increase in HL overall (Supplementary Fig. S1). Accordingly, infected individuals had higher HL than uninfected individuals overall and in most subspecies (Fig. 2a,b). Subspecies was also an important predictor of infection status itself: the probability of infection was higher in *P. e. elegans* and *P. e. flaveolus* than in *P. e. adelaidae* and the Western Slopes population (Table 2; Supplementary Fig. S1). Additionally, infection probability increased with age, with subadults having a higher probability of infection than young or old adults (Table 2). Host sex appeared in the second and third ranked models, but the confidence intervals for the sexes were overlapping. There was no evidence that average pairwise relatedness (APR) was associated with infection status (Tables 1 and 2). To assess whether APR and HL may have confounding effects on BFDV infection probability, we added APR to the top model shown in Table 1. When APR was added to the top model containing subspecies, age and HL, the delta AIC_c_ was 2.14 and the 95% CI estimate for APR overlapped zero (estimate ± SE = −0.21 ± 3.27, 95% CI = −6.62 to 6.21), while the 95% CI of parameter estimates for HL remained non-overlapping zero (estimate ± SE = −2.44 ± 1.18, 95% CI = −4.75 to −0.13). This showed that whilst controlling for APR, HL remains an important predictor of BFDV infection. We found no evidence to suggest that local genetic effects explained the effect of HL on BFDV infection probability (N = 219; F = 1.122, df = 8, P = 0.350).

**Table 1:** Models considered plausible (Akaike weight > 0.05) for predicting the effect of host traits in *Platycercus elegans* on (a) beak and feather disease virus infection status, and (b) viral load; n = 224, tests of viral load (i.e. relative viral gene expression) were based on the subset of individuals that were infected with BFDV (n = 106). Addition sign represents delta AIC_c_ compared to top model.

Ranked models	AIC_c_	*w*	Cumulative *w*	Model likelihood	Evidence ratio
*(a) BFDV infection status*					
**HL + subspecies + age**	**222.54**	**0.38**	**0.38**	**1**	—
**HL + subspecies + age + sex**	**+0.59**	**0.29**	**0.67**	**0.74**	**1.34**
Subspecies + age + sex	+2.22	0.13	0.80	0.33	3.03
Subspecies + age	+2.51	0.11	0.91	0.28	3.51
*(b) BFDV viral load*					
**APR + subspecies + age + sex**	**414.97**	**0.53**	**0.53**	**1.00**	—
Subspecies + age + sex	+1.56	0.25	0.78	0.46	2.18
**HL + subspecies + age + sex**	**+3.80**	**0.08**	**0.86**	**0.15**	**6.67**
**APR + subspecies + age**	**+4.32**	**0.06**	**0.92**	**0.12**	**8.66**

APR = average pairwise relatedness, HL = homozygosity-by-loci, AIC_c_ = Akaike Information Criterion (corrected for small sample size), *w* = Akaike model weight. Model likelihood is the relative likelihood of each model compared to the top ranked model; evidence ratio is how much less likely each model is than the top ranked model. Bold indicates models containing genetic diversity variables.

**Table 2:** Model averaged parameter estimates and parameter weights for the effect of host traits in *Platycercus elegans* on (a) beak and feather disease virus infection status (infected or uninfected), and (b) viral load (viral gene expression, log_10_). The set of candidate models included all combinations of one or more predictors, except HL and APR which were not included together because their correlation may bias estimates.

Parameter		Estimate/odds-ratio	SE	Lower 95% CI	Upper 95% CI	Parameter weight
***(a)*** ***Infection status***						
Subspecies*	*P. e. elegans*	0.79	0.06	0.66	0.91	>0.99
	Western Slopes	0.22	0.06	0.11	0.33	
	*P. e. flaveolus*	0.87	0.07	0.73	>0.99	
	*P. e. adelaidae*	0.36	0.06	0.24	0.48	
Age*	Subadult	0.79	0.06	0.68	0.91	>0.99
	Young adult	0.52	0.09	0.34	0.69	
	Old adult	0.40	0.08	0.25	0.55	
HL**		**10.79**		**1.11**	**105.21**	**0.67**
Sex*	Male	0.65	0.07	0.51	0.79	0.46
	Female	0.52	0.07	0.38	0.66	
APR**		4.30		0.01	2090.15	0.09
***(b) Viral load***						
Subspecies	*P. e. elegans*	−0.67	0.29	−1.23	−0.11	>0.99
	Western Slopes	−3.06	0.45	−3.95	−2.18	
	*P. e. flaveolus*	−0.72	0.35	−1.4	−0.03	
	*P. e. adelaidae*	−2.64	0.33	−3.29	−1.99	
Age	Subadult	−0.99	0.23	−1.45	−0.53	0.96
	Young adult	−2.15	0.36	−2.86	−1.44	
	Old adult	−2.20	0.35	−2.88	−1.52	
Sex	Male	−1.36	0.23	−1.82	−0.90	0.89
	Female	−2.20	0.26	−2.70	−1.69	
**APR**		**6.00**	**2.98**	**0.16**	**11.85**	**0.63**
HL		0.48	0.5	−0.51	1.46	0.10

APR = average pairwise relatedness, HL = homozygosity-by-loci, OR = odds-ratio, SE = standard error, CI = confidence interval. *For the binary response variable (BFDV infection status), parameter estimates for categorical variables (subspecies, age, sex) represent the proportion of infected individuals in each group (±SE). ** For the binary response variable (BFDV infection status), parameter estimates for continuous variables (HL, APR) represent the odds-ratio (increased likelihood of infection). Bold indicates continuous variables for which the 95% confidence interval does not span one (for odds-ratios) or zero (for estimates).

### Predictors of BFDV viral load

Four plausible models were found to predict viral load, with a combined likelihood of 92.1% (Table 1). The best model was 1.2 × 10^10^ times more likely than the null model (intercept only). As with infection status, all of these models included the predictors subspecies and age class. In contrast to infection status, in models predicting viral load APR appeared in the top-ranked model as well as the fourth ranked model, while HL only appeared in the third ranked model (Table 1). Based on parameter weights, APR was ranked the fourth most important parameter after subspecies, age and sex in predicting viral load (Table 2). APR was positively associated with viral load in all subspecies (Table 2; Fig. 2c,d). Viral load was higher in *P. e. elegans* and *P. e. flaveolus* than in *P. e. adelaidae* and the Western Slopes population (Table 2). Young and old adults had similar levels of viral load, and both were lower than subadults (Table 2). Host sex was a well-supported predictor of viral load, appearing in the top three plausible models (Table 1). However, while males tended to have higher viral load than females, the 95% confidence interval for the sexes was overlapping (Table 2). HL was not associated with viral load, having low parameter weight and an odds-ratio confidence interval that overlapped one (Table 2). To assess whether APR and HL may have confounding effects on BFDV load, we added HL to the top model shown in Table 1. When HL was added to the top model containing subspecies, age, sex and APR, the delta AIC_c_ was 2.4 and the 95% CI estimate for HL overlapped zero (estimate ± SE = −0.22 ± 1.03, 95% CI = −2.25 to 1.81), while the 95% CI of parameter estimates for APR remained non-overlapping zero (estimate ± SE = 6.07 ± 3.07, 95% CI = 0.05 to 12.09). This showed that when controlling for HL, APR remained an important predictor of BFDV viral load. We found no evidence to suggest that local genetic effects explained the effect of HL on BFDV viral load probability (N = 219; F = 1.118, df = 8, P = 0.352).

### Genetic diversity across subspecies

HL was significantly different between the subspecies and WS populations (ANOVA; F = 8.30, df = 3, P < 0.001). *P. e. elegans* had a lower HL (greater heterozygosity) compared to all other subspecies, whilst *P. e. adelaidae* was more heterozygous than WS (Fig. 1b). APR was not significantly different between the subspecies (ANOVA; F = 1.79, df = 3, P = 0.15), although *P. e. flaveolus* tended to be lower (Fig. 1c). Genetic diversity was not significantly different between the subspecies in terms of allelic richness (ANOVA; *F* = 0.49, df = 3, P = 0.69; Fig. 1d), but private allelic richness did vary significantly (ANOVA; F = 3.40, df = 3, P = 0.03; Fig. 1e), although both showed the same general patterns between subspecies. In general, *P. e. elegans* and *P. e. flaveolus* populations represented the extremes of genetic diversity, and thus there were no obvious associations between population genetic diversity and population differences in the prevalence or average load of BFDV infection.

### Host and BFDV genetic distance

When comparing host and virus genetic distances between individuals, whilst controlling for geographic distance, we found no correlation between BFDV genetic distance and host genetic distance (Partial BFDV genome: Mantel r = 0.01, n = 36, *P* = 0.85; REP: Mantel r = 0.003, n = 26, *P* = 0.94; CAP: Mantel r = −0.09, n = 31, *P* = 0.05). Removing sequences that showed evidence of recombination did not alter these results (Partial BFDV genome: Mantel r = 0.07, n = 25, *P* = 0.20; REP: Mantel r = 0.11, n = 16, *P* = 0.25; CAP: Mantel r = 0.01, n = 22, *P* = 0.84).

## Discussion

In this study, we tested whether individual heterozygosity (HL) and genotype rarity (APR) predicted infection status and viral load of beak and feather disease virus (BFDV) in *P. elegans*. Our analyses revealed that HL was an important predictor of infection status, as expected from similar studies in other host-pathogen systems, but that APR was not. In contrast, for viral load our results indicated that APR was an important predictor, but HL was not. Our analyses controlled for other host variables including subspecies, age class and sex, and supported previous findings^22^ in showing that subspecies, followed by age class, were also important predictors of BFDV infection status and load in this host system^22^. We further showed that genetic diversity varied between populations, but not in a direction consistent with population level differences in BFDV prevalence or load, and that host genetic distance was not significantly correlated with virus genetic distance across populations.

A key finding of our study is that individuals with lower heterozygosity (higher HL) were more likely to be infected with BFDV (Fig. 2a). This pattern was most clearly observed in *P. e. elegans* and *P. e. adelaidae* (Fig. 2b); however, the available data suggest there were no significant interactions between any predictors including subspecies and HL. The apparent absence of an effect of HL on BFDV infection in *P. e. flaveolus* and the WS population could potentially be due to the smaller sample sizes and skewed prevalence (high and low respectively). A positive relationship between individual host heterozygosity and infection status has been found in studies on other host-pathogen systems, often using a moderately sized panel of microsatellite loci (compared to genome-wide sequencing studies) as we did in this study^5,6,8–10,15,16^. Similarly, our effect sizes are within a similar range to previous work on host heterozygosity and infection^16^. Taken together, our study in concert with previous findings, suggest that host heterozygosity offers a modest but significant advantage in terms of reducing susceptibility to infection in general. The specific mechanisms underlying this relationship remain to be determined, but might relate to the neutral markers reflecting multi-locus genetic diversity across the whole genome, and therefore, potentially across immune effector loci, or loci with epistatic effects on resistance/tolerance. Individuals that have higher levels of heterozygosity may be able to resist a wider range of pathogens, or in this instance a greater range of BFDV variants. Although a single individual can host several different genetic strains, BFDV is relatively conserved anti-genetically^29^. Alternatively, higher levels of heterozygosity may reflect an immunogenic advantage due to overdominance or due to rare allele advantage. In contrast, less heterozygous individuals are more likely to display deleterious recessive alleles as a result of inbreeding depression, resulting in increased pathogen susceptibility^15^.

We also found that multilocus genotype rarity, unlike heterozygosity, was an important predictor of BFDV load among the 106 BFDV infected hosts. APR showed a positive association with viral load, a pattern which was evident in all subspecies and the WS population (Fig. 2c,d). Thus, our results indicate that the more related an individual was to others within its population, the more likely it was to harbour a higher infection load than less related infected individuals. We speculate that this finding may be the result of viral tracking of common host genotypes, whereby viral genotypes co-evolve with host susceptibility. BFDV variants that have co-evolved with common host genotypes may be more compatible with, and more able to replicate in, hosts harbouring these genotypes^17–19^. This would give rare host genotypes within a population a selective advantage, potentially leading to a negative frequency-dependent scenario (i.e. ‘Red Queen’ dynamics)^18^. However, when comparing pairwise host and pairwise virus genetic distances between individuals whilst controlling for geographic distance, we found no significant correlations between BFDV genetic distance and host genetic distance. That suggests that BFDV may not be strongly co-adapted to the most common host genotype, as we might have expected given BFDV viral load was found to be higher in common host genotypes. However, differences in evolutionary rates between the host and pathogen, or alternatively, sampling at too high spatial and/or temporal scale are plausible explanations for this negative finding^35^. Furthermore, BFDV genetic distance and host genetic distance are measured at the individual level, which may not reflect pathogen adaptation to common genotypes at the population level. Future research is needed to explore coevolution across multiple generations to test for “Red Queen” dynamics. The finding that individual birds with more common genotypes had a higher BFDV load could also provide an important explanation for the evolution of host dispersal; individuals that possess a common genotype would be under more intense pressure to disperse in order to escape co-adapted pathogens^9^. Our results could provide support for a disease escape-by-dispersal hypothesis, because it is likely that our average pairwise relatedness measure is correlated with dispersal between populations^36^.

At the subspecies level, if pathogen infections select against high levels of host homozygosity, then we might expect that populations under greater pathogen selective pressure would be more heterozygous^2^, or alternatively, more homozygous populations could be more susceptible and therefore display a higher prevalence. However, when there are multiple comparisons across different populations it is difficult to determine causality in heterozygosity-fitness correlations^37^. Although we found that genetic diversity varied between subspecies (Fig. 1), genetic diversity differences between subspecies did not appear to be related to the prevalence of BFDV infection at a population level (Table 2). These results may be a consequence of a broader range of selective and stochastic forces or differences in population size^38^.

For studies examining heterozygosity-fitness correlations, it is important to utilise a set of neutral markers that reflect genome-wide heterozygosity. Our analyses (g_2_, tests of single loci) indicated that the markers we used do reflect heterozygosity across the genome and the number of markers we have used is within a similar range to other studies detecting heterozygosity-fitness correlations^39,40^. Increasing the number of markers used in this analysis is likely to improve the estimates as demonstrated in Hoffman *et al*.^11^. However, it may also be useful to examine specific functional regions such as the major-histocompatibility complex^12,13^, Toll-like receptors^14^ or cytokine regions^41^, which may improve our understanding of the mechanism through which heterozygosity affects fitness.

One potential confound in our approach to investigating the role of genotype rarity in infection is that rare alleles are more likely to occur in heterozygous individuals (i.e. because they are rare, they are unlikely to occur twice in the same individual). However, HL and APR were only weakly correlated and the distribution of APR in groups of high and low HL was similar (Supplementary Figs S2 and S3), suggesting that both measures are largely independent. The association between heterozygosity and rare alleles may not translate into the whole genome level as measured using microsatellites. Furthermore, instances where common heterozygotes or homozygotes with rare alleles exist (e.g. immigrants) are likely to separate the two metrics. However, we note that ours is the first use of APR as a measure of genotype rarity and further validation of the metric is needed to investigate the effectiveness of this approach.

In conclusion, using wild populations of a species complex, we show that a novel measure of an individual’s genotype rarity (APR) predicts pathogen load across subspecies. Our findings also support the hypothesis that individual heterozygosity is negatively associated with infection probability (i.e. greater heterozygosity associated with lower probability of infection), in line with previous similar studies in a range of host-pathogen systems^5,6,8–10,15,16^. Our results suggest that an individual’s infection susceptibility and severity may be under different selection regimes: homozygous hosts may be selected against due to increased pathogen susceptibility and therefore would be under negative directional selection^2^. However, host and pathogen genotypes may also be under negative frequency-dependent selection, with common genotypes suffering a greater infection severity^17,21^. We provide evidence that both pathogen-mediated mechanisms for explaining the maintenance of host genetic diversity can operate concurrently. Our work offers novel support to the view that pathogens may be an important factor in host genetic divergence^22^. Our findings also provide insight into the genetic determinants of the spread of infection in natural populations. The patterns that we have uncovered may have implications for the evolution of dispersal, mate choice decisions by hosts, and for conservation management involving pathogens in small, fragmented populations.

## Methods

### Fieldwork

During 2004–2011, *P. elegans* (n = 224) were sampled throughout the range of the species in south-eastern Australia, encompassing the three mainland subspecies (*P. elegans elegans* n = 53, *P. e. flaveolus* n = 30, and *P. e. adelaidae* n = 76) and a putative hybrid population (‘Western Slopes, WS’) which occurs where *P. e. elegans* and *P. e. flaveolus* overlap on the western slopes of the Great Dividing Range (n = 65; Fig. 1a)^27^. Sampling sites and times were the same as described elsewhere^22^. *P. e. melanoptera*, which is a small, geographically isolated population on Kangaroo Island (Fig. 1a)^27^, was not sampled for this study. Moreover, we only sampled *P. elegans* from south of the Hunter River in NSW (i.e. south of S32°56′), as previous microsatellite and mitochondrial DNA analyses have shown that birds north of this location represent a genetic cluster distinct from all southern populations^27^. Blood or tissue samples were collected and used for genotyping, molecular sexing, and BFDV testing following Eastwood *et al*.^42^. The age class of each individual was scored based on distinct plumage characteristics, following Eastwood *et al*.^42^: green body plumage denoted sub-adult (1^st^ year birds); yellow-red body plumage with a white underwing stripe denoted “young adults” (2^nd^ and 3^rd^ year birds); yellow-red body plumage and no white underwing stripe denoted “old adults” (>3 years). All work involving animals was approved by Deakin University’s Animal Ethics Committee (Project no: A33-2008 and A51-2011), and in accordance with the legal requirements of Australia and the relevant states.

### Molecular techniques

Ammonium acetate DNA extraction was used for all samples^42^ and sex was determined using molecular methods^43^. To measure BFDV prevalence and load, we first standardised DNA concentration with a Beckman DU spectrophotometer (Beckman Coulter, CA, USA) and then used a probe based quantitative real-time PCR technique, which has previously been used^22^ and validated in *P. elegans* ^42^. For full details of BFDV detection in this species see Eastwood *et al*.^42^. In brief, the method amplifies a 100 nucleotide region in the highly conserved replication-associated protein ORF using the following primer set: forward primer 900 nm (5′-GAC GCG GAT AAC GAG AAG TAT TG-3′), reverse primer 300 nm (5′-GCA ACA GCT CCA TCG AAA GC-3′) and probe 100 nm (5′-FAM CCG TCT CTC GCC ACA ATG CCC AGG TAMRA-3′). Quantitative PCR was performed in a Stratagene Mx3005 P (Agilent Technologies, CA, USA) with Brilliant Multiplex qPCR master mix solution (Agilent Technologies, USA). We used the following qPCR conditions: initial denaturation of 10 min at 95 °C; followed by 40 cycles of: 30 s at 95 °C, 60 s at 60 °C and 30 s at 72 °C; followed by final extension of 5 min at 72 °C. The method is highly repeatable (inter-assay r = 0.99) and reproducible between repeated extractions (r = 85). Both blood and muscle tissue samples were used in this study which give highly similar viral load estimates^42^. For BFDV positive samples (n = 106), BFDV load was measured as relative gene expression using the comparative Ct method^44^ and relative to a control sample (BFDV positive individual that was confirmed by sequencing)^22,42^.

### Microsatellite genotyping

Rosella samples were genotyped using nine microsatellite loci (*AgGT07, AgGT21*, *CP03E01*, *CP52A09*, *Ero03*, *Ero08*, *Cl2*, *Cl3*, and *Cfor2627*), using the methods described in Joseph *et al*.^27^. In addition to the n = 224 samples collected, aged, sexed and tested for BFDV for this study as described above, we added a further n = 139 for which genotypes using the same microsatellite methods were already known^27^; this larger dataset was used for tests of null alleles, linkage disequilibrium, Hardy-Weinberg equilibrium, and population differences in genetic diversity parameters (see Supplementary Information). Sample sizes for the combined dataset were as follows: *P. e. elegans*: n = 100; *P. e. flaveolus*: n = 52; WS: n = 99; *P. e. adelaidae*: n = 112. Using IRmacroN3 (W. Amos, Cambridge University) implemented in Microsoft Excel 2010, we determined the number of alleles and the allele size ranges per locus, and estimated the proportion of null alleles. Using Arlequin version 3.5, we found no deviations from Hardy-Weinberg equilibrium or linkage equilibrium and low F_ST_ values which indicate little population differentiation, (see Supplementary Tables S1, S2 and S3)^45^. To test whether our panel of microsatellites can reflect genome-wide heterozygosity we calculated g_2_^46^ using the R package InbreedR^47^. The g_2_ statistic was positive but did not significantly depart from zero using nine microsatellite loci from 363 individuals (g_2_ = 0.003 ± 0.006, 95% CI −0.009 to 0.015, *P* = 0.36, 1000 permutations). To test the strength of single locus effects on our g_2_ statistic, we performed a delete-one jackknife which revealed that the exclusion of two loci, C13 and Cfor2627, resulted in a more positive g_2_ statistic (Δg_2_ 0.004 and 0.002, respectively), while the removal of any of the other seven loci had a negative influence on our g_2_ statistic (mean Δ g_2_ −0.004, range −0.006 to −0.001). Subsequently, we excluded both C13 and Cfor2627 which resulted in a g_2_ statistic significantly greater than zero (g_2_ = 0.013 ± 0.007, 95% CI −0.001 to 0.03, *P* = 0.03, 1000 permutations), suggesting that this reduced panel of seven loci may better reflect genome-wide heterozygosity than the full panel of nine loci. However, there was a strong correlation between HL calculated from the full panel and from the reduced panel of seven loci (Spearman’s *r* = 0.88, P < 0.001), and both sets of loci gave similar conclusions regarding the effect of heterozygosity when used interchangeably in the top models (GLM including HL calculated using nine loci: estimate = −2.46, standard error = 1.15, lower 95% CI = −4.72, upper 95% CI = −0.20; GLM including HL calculated using seven loci: estimate = −2.37, standard error = 1.01, lower 95% CI = −4.34, upper 95% CI = −0.40). The g_2_ values in this study, using either nine or seven microsatellites, are similar to those reported in other studies investigating heterozygosity fitness correlations^48^, and a g_2_ significantly different from zero should not be considered a pre-requisite for testing heterozygosity fitness correlations^48,49^. For these reasons, we used all nine loci for the analyses reported elsewhere in this study.

### Genetic diversity parameters

We calculated measures of genetic diversity of hosts at both the individual and population levels. At the individual level, all metrics were calculated based on allele frequencies within each subspecies. Homozygosity-by-loci (HL) was calculated using the excel macro IRmacroN3, as described in^50^. HL estimates the level of inbreeding within an individual (homozygosity) by weighting the allelic contribution of each locus. More homozygous individuals have an HL value closer to one, and more heterozygous individuals a value closer to zero. To estimate the rarity of an individual’s genotype within the population, we calculated average pairwise relatedness (APR) to all other individuals sampled within the population. This simple measure of genotype rarity is based on the assumption that a common genotype will have a higher average pairwise relatedness compared to a rare genotype in the same population. To calculate pairwise relatedness to all individuals, we used the method described by Queller and Goodnight^51^ implemented in Coancestry (Version 1.0.1.7)^52^. To provide some additional validation that APR is a robust, general estimate of genotype rarity we quantified the probability of belonging to a population using a genetic assignment method implemented in Geneclass2^53^. Typically used for identifying dispersers, Geneclass2 calculates a likelihood estimate (Likelihood computation used = L_home; we treated subspecies as independent populations)^54^; using Monte Carlo re-sampling, the program simulates a random sample of expected genotypes (n = 10,000). This calculates the probability that an individual’s genotype is found within a given population^54^, hereafter the probability of assignment index. APR and the probability of assignment index were highly correlated (Spearman’s r = 0.76, P < 0.001) suggesting that APR has the ability to distinguish between dispersers, common genotypes and mixed genotypes (Supplementary Fig. S2). In addition, we investigated the relationship between APR and HL because they may be correlated (i.e. rare alleles are more likely to be present in heterozygous individuals). HL was correlated with APR (Spearman’s r = 0.26, P < 0.001) but only explained 8 percent of the variance. HL was not correlated with the probability of assignment index (Spearman’s r = 0.04, P = 0.56). In addition, we assessed the frequency distribution of APR in two groups (data split in two groups by the mean: high HL and low HL) which would identify if APR was biased according to heterozygosity. However, this was not the case; see Supplementary Fig. S3. The overall range of the individual genetic diversity parameters was large (HL: 0–0.896, APR: −0.179–0.140). The distribution of all individual-level genetic diversity parameters were broadly similar in all subspecies (see Supplementary Fig. S4). Following Rollins *et al*.^55^, we tested for any population sub-structure that may bias our individual level metrics using the program Structure (version 2.3.4). The results indicated that there was no population sub-structure at the subspecies level (data not shown). This analysis concurred with earlier findings in *P. elegans* which reported four homogenous genetic clusters corresponding to the three subspecies and the WS population based on microsatellite data^27^.

Following Szulkin *et al*.^49^, we tested for genetic local effects on heterozygosity by analysing whether the models containing HL explained less variance than the models including single-locus heterozygosities, which were assigned as either heterozygous or homozygous at each locus separately (n = 9 loci). Single-locus heterozygosity variables or HL were included separately as fixed predictors in the top model for BFDV infection including subspecies and age, and the top model for BFDV viral load including subspecies, age and sex. For each comparison, BFDV infection and BFDV viral load, the residual deviances from both models (Single-locus heterozygosity variables or HL) were compared using an F-ratio test.

At the population level, genetic diversity was estimated using an additional two parameters, allelic richness (which estimates the average number of alleles within a population) and private allele richness (which estimates the average number of unique alleles within a population). Both were calculated with the program HP-rare version 1^56^ using the rarefaction method^57^.

### Host distance versus BFDV distance

For a subsample of infected samples (n = 36), BFDV sequences were available (GenBank accession numbers KJ953847 - KJ953885)^22^. These data were used to test for correlations between *P. elegans* and BFDV genetic distance while controlling for geographic distance, using partial Mantel tests (as implemented in XLSTAT, version 2014.02.03, Addinsoft). Genetic distance in *P. elegans* was calculated by transforming the Queller and Goodnight^51^ relatedness coefficient for each dyad by subtracting this value from one. Genetic distances range between zero (high similarity) and two (very distant). Pairwise BFDV genetic distance, recombination detection, and geographic distance were calculated following Eastwood *et al*.^22^. We used three different BFDV genome segments for analysis, which correspond to different coding regions that may be under different selective pressures. These included a 726 nucleotide segment of the replication associated open reading frame (Rep ORF), a 744 nucleotide segment of the capsid ORF (Cap ORF), and lastly, these regions concatenated with a non-coding region, hereby termed partial BFDV genome (1629 nucleotides). Mantel tests were repeated after excluding sequences with evidence for recombination^22^, with similar results obtained.

### Statistical analysis

To identify the best predictors of BFDV infection status (infected/uninfected) and viral load (relative viral gene expression; infected birds only; log_10_ transformed to achieve normality), we used model selection based on Akaike Information Criteria (corrected for small sample size; AIC_c_). Using generalized linear models (GLM), we compared the same set of candidate models for infection status (binomial probability distribution and logit link function) and viral load (normal probability distribution and identity link function). The predictors were host traits that have previously been identified as important in BFDV (subspecies, age class and sex)^20^ as well as the two individual genetic diversity measures hypothesised to influence infection (HL and APR); all predictors were modelled as fixed effects. The set of candidate models included all combinations of one or more predictors, except that the two genetic diversity measures were not included together in models in case their correlation biased estimates. This decision to analyse HL and APR separately was done *a priori*, however we additionally tested for confounding effects between HL and APR by including these terms together in the top models of both BFDV infection and viral load. The final candidate set included 23 models for each response variable, which are shown in Supplementary Table S4. In addition, we included intercept only models for each response variable (null model). Models were ranked by AIC_c_ weight, and models were considered plausible if their AIC_c_ weight was >0.05.

To calculate robust estimates of effect sizes, we carried out model averaging of parameter estimates and errors, following Symonds and Moussalli^58^. We averaged over the models in which each parameter of interest appears, using model weights renormalized to sum up to one, to obtain biologically relevant estimates of how each predictor related to the response variables^58^. For the binary response variable (infection status), parameter estimates for categorical variables (subspecies, age, sex) indicate the proportion of infected individuals in each group, while for continuous variables (HL, APR) the parameter estimates indicate the odds-ratio (i.e. increased likelihood of infection from a one unit increase in the predictor). For the continuous response variable (viral load), all estimates indicate the slope. Continuous predictors (HL and APR) were considered important (or ‘significant’) if the 95% confidence range of model averaged estimates did not overlap one (for odds-ratios) or zero (for slopes). Categorical predictors (subspecies, age class, sex) were considered important if the model averaged 95% confidence ranges of their groups did not overlap or parameter weight was >0.95. Parameter weights were calculated by summing the weights for all models in which that parameter appears, to provide an estimate of the relative importance of each predictor.

We evaluated interactions between genetic diversity and subspecies, and between host sex and age, using a post-hoc approach. Using the top model for both BFDV infection and BFDV viral load model selection analyses, we included the interaction between subspecies and either HL or APR. However, all interactions using subspecies and a genetic measure were non-significant (P > 0.05) and no interactions led to more strongly supported top models for infection status and viral load (based on ΔAIC_c_). Likewise, we tested whether there was an interaction between age and sex within the top models for both BFDV infection and viral load. The interaction between age and sex was non-significant in both cases (P > 0.05) and was therefore not included in the final analyses.

Data were examined to ensure the assumptions of each statistical analyses were met; viral gene expression was not normally distributed and this was rectified using log transformation prior to modelling. Statistical analyses were performed using the programs R^59^ and SPSS version 24 (IBM, Armonk NY). Means and estimates are shown with standard error unless otherwise stated.

## Electronic supplementary material

Supplementary Information
