# Supplementary material for: Host heterozygosity and genotype rarity affect viral dynamics in an avian subspecies complex
Source: Sci Rep. 2017 Oct 17;7:13310. doi: 10.1038/s41598-017-13476-z (PMC5645371; doi:10.1038/s41598-017-13476-z)
Supplement: Supplementary file 1 — Supplementary Information [file 41598_2017_13476_MOESM1_ESM.pdf]

ONLINE SUPPLEMENTARY INFORMATION

Host heterozygosity and genotype rarity affect viral dynamics in an avian subspecies complex

Authors:

Justin R. Eastwood[1, 2], Raoul F. H. Ribot[1], Lee Ann Rollins[1], Katherine L. Buchanan[1], Ken Walder[3], Andrew T. D. Bennett[1], Mathew L. Berg[1]

Affiliations:

[1] Centre for Integrative Ecology, School of Life and Environmental Sciences, Deakin University, Geelong 3216, Victoria, Australia.

[2] School of Biological Sciences, Monash University, Melbourne, Victoria, 3800, Australia.

[3] Metabolic Research Unit, School of Medicine, Deakin University, Geelong 3216, Victoria, Australia.

Corresponding author: justin.eastwood@monash.edu.au

Supplementary Table S1: F_{st} values between each subspecies dyad calculated using Alequin.

Subspecies	*P. e. elegans*	Western slopes hybrid	*P. e. flaveolus*	*P. e. adelaidae*
P. e. elegans	0.00	-	-	-
Western slopes hybrid	0.03	0.00	-	-
P. e. flaveolus	0.07	0.04	0.00	-
P. e. adelaidae	0.02	0.05	0.06	0.00

Supplementary Table S2: Summary statistics of each loci used in this study assuming all subspecies are one continuous population. Each loci was tested for linkage disequilibrium within each subspecies. Loci C12 had a high proportion of null alleles which ranged between 0.10 and 0.17 across subspecies. Excluding C12 from the analysis did not influence the results of this study.

Loci	Number of individuals	Number of alleles per locus	Allele size range	Estimated proportion of null alleles	Evidence of linkage disequilibrium?
AgGT07	359	27	251-286	0.1	No
Cp03E01	361	23	184-234	0.1	No
AgGT21	361	4	315-328	0.02	No
Cfor2627	362	10	132-159	0.04	No
C12	362	6	61-78	0.17	No
C13	363	8	189-209	0.09	No
CP52A09	359	20	361-401	0.04	No
Ero03	358	12	192-218	0.06	No
Ero08	362	17	119-155	0.01	No

Supplementary Table S3: Arlequin results for observed and expected heterozygosities used for testing Hardy-Weinberg equilibrium for each subspecies (n = 363 individuals) at each of the nine microsatellite loci. * denotes significance after Bonferroni correction ($P < 0.001$).

**Loci**	**Heterozygosity**	***P. e. elegans***	**Western Slopes**	***P. e. flaveolus***	***P. e. adelaidae***	**Pooled data**
**AgGT07**	H_o	0.83	0.73	0.77	0.71*	0.75*
	H_e	0.93	0.85	0.88	0.86	0.92
**CPO3E01**	H_o	0.86	0.49*	0.59	0.86	0.72*
	H_e	0.90	0.88	0.81	0.88	0.89
**AgGT21**	H_o	0.43	0.33	0.17	0.32	0.33
	H_e	0.41	0.35	0.20	0.31	0.34
**Cfor2627**	H_o	0.63	0.63	0.54	0.5	0.58
	H_e	0.69	0.67	0.59	0.52	0.63
**Cl2**	H_o	0.40	0.29	0.27	0.40	0.35*
	H_e	0.52	0.40	0.34	0.50	0.50
**Cl3**	H_o	0.55	0.58	0.58	0.51*	0.55*
	H_e	0.61	0.63	0.67	0.59	0.65
**CP52A09**	H_o	0.78	0.73	0.77	0.79	0.77*
	H_e	0.81	0.87	0.84	0.82	0.84
**Ero03**	H_o	0.70	0.60	0.55	0.66	0.64*
	H_e	0.73	0.64	0.66	0.75	0.72
**Ero08**	H_o	0.87	0.81	0.65	0.73	0.78*
	H_e	0.84	0.82	0.60	0.76	0.80

Supplementary Table S4: The set of candidate models considered in this study, ranked by the AIC_c values for each of the two response variables (lower AIC_c values indicates greater support for model). Addition sign represents delta AIC_c compared to top model.

**Response variable**	**Candidate models**	**AIC_c**
BFDV infection status	Subspecies + Age + HL	222.54
	Subspecies + Age + Sex + HL	+0.59
	Subspecies + Age + Sex	+2.22
	Subspecies + Age	+2.51
	Subspecies + Age + Sex + APR	+4.21
	Subspecies + Age + APR	+4.35
	Subspecies + HL	+11.94
	Subspecies + Sex + HL	+14.05
	Subspecies	+14.72
	Subspecies + APR	+15.99
	Subspecies + Sex	+16.7
	Subspecies + Sex + APR	+17.98
	Age + Sex	+37.14
	Age + Sex + APR	+38.39
	Age + Sex + HL	+38.95
	Age	+40.36
	Age + APR	+41.67
	Age + HL	+41.74
	Null (intercept only)	+89.37
	HL	+90.46
	Sex	+90.66
	APR	+91.16
	Sex + HL	+91.99
	Sex + APR	+92.47
BFDV viral load	Subspecies + Age + Sex + APR	414.97
	Subspecies + Age + Sex	+1.56
	Subspecies + Age + Sex + HL	+3.8
	Subspecies + Age + APR	+4.32
	Subspecies + Age	+5.79
	Subspecies + Sex + APR	+6.15
	Subspecies + Age + HL	+7.29
	Subspecies + APR	+8.75
	Subspecies + Sex	+11.01
	Subspecies + Sex + HL	+12.8
	Subspecies	+13.3
	Subspecies + HL	+14.31
	Age + Sex	+23.23
	Age + Sex + HL	+25.01
	Age + Sex + APR	+25.32
	Age	+28.88
	Age + APR	+30.99
	Age + HL	+31.07
	Sex	+43.99
	Sex + APR	+44.96
	Sex + HL	+45.96
	Null (intercept only)	+46.41
	APR	+47.51
	HL	+48.53

APR = average pairwise relatedness, HL = homozygosity-by-loci, AIC_c = Akaike Information Criterion (corrected for small sample size)

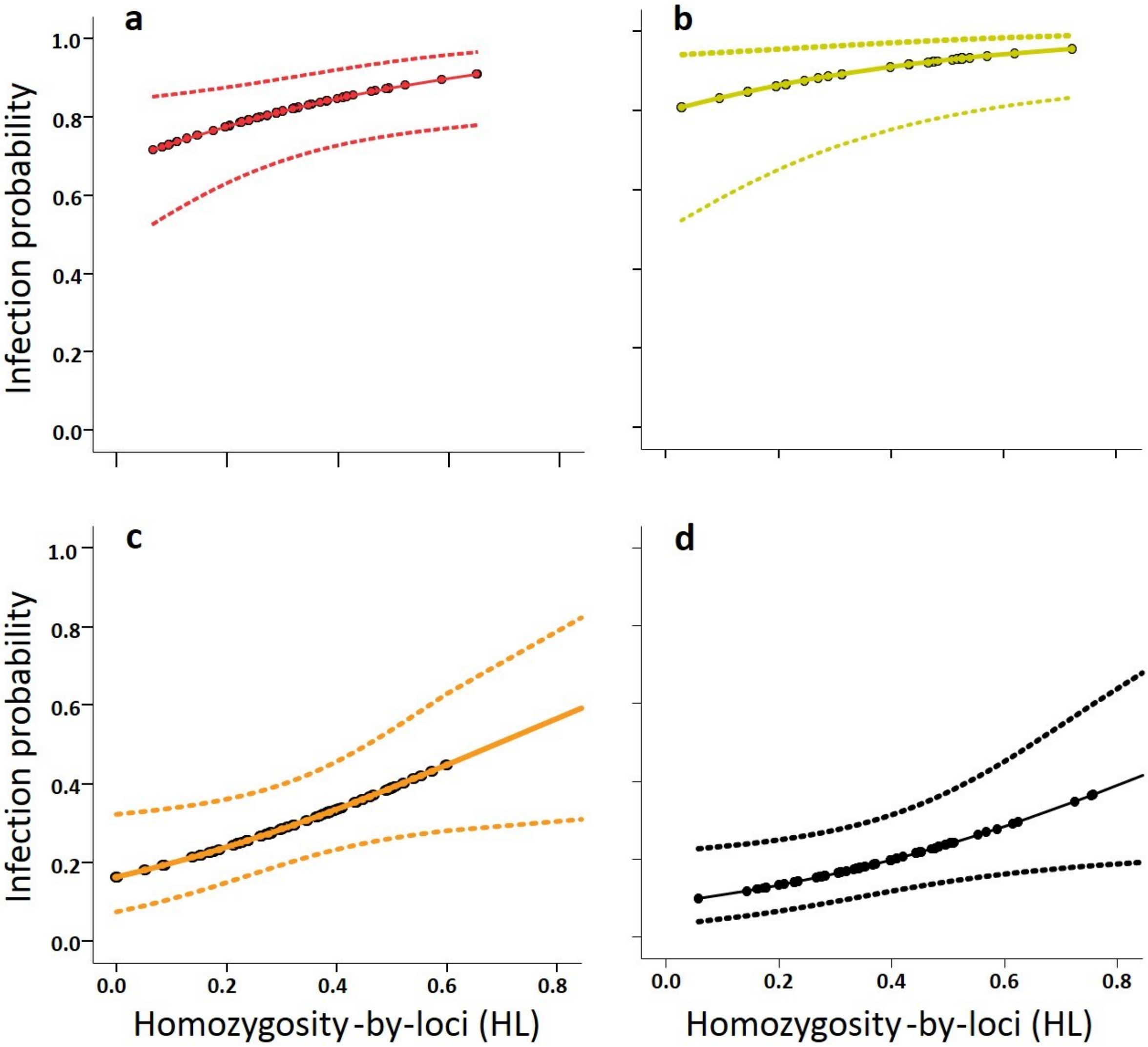

Supplementary Figure S1: The model predicted effects of individual host homozygosity (measured as homozygosity-by-loci) on the probability of infection with beak and feather disease virus in **(a)** *Platycercus elegans elegans*, **(b)** *P. e. flaveolus*, **(c)** *P. e. adelaidae*, and **(d)** the Westerns Slopes population. Dotted lines indicate the 95% confidence interval, and circles indicate the levels of HL sampled in this study. Effects are averaged over age classes and sexes.

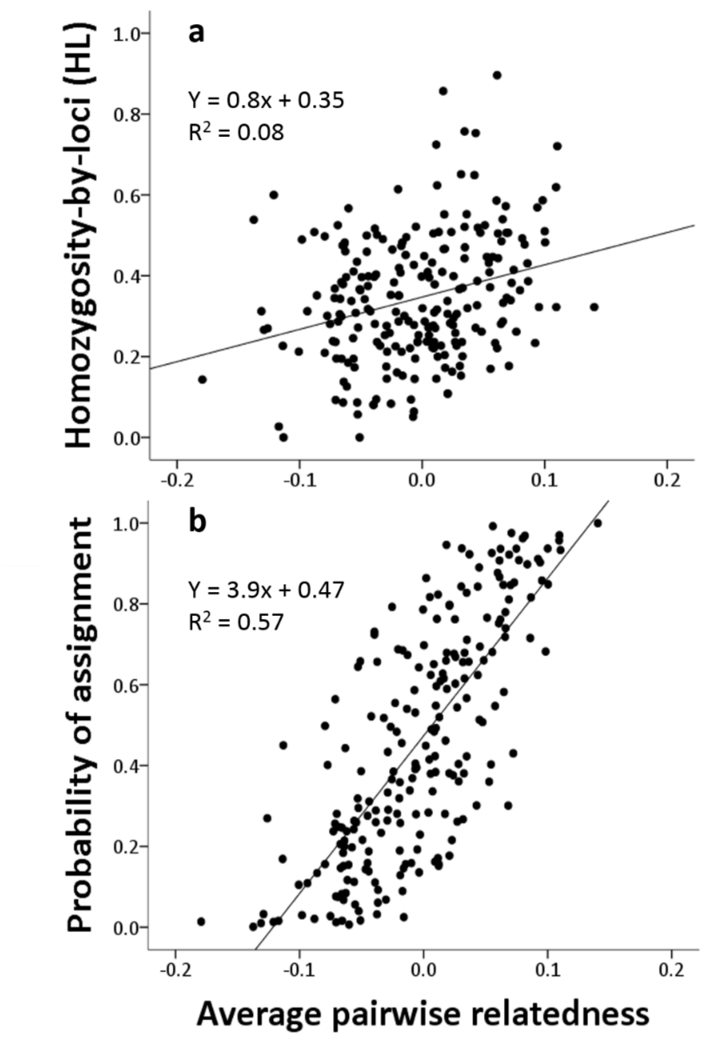

Supplementary Figure S2: To estimate an individual's genotype rarity within a population we calculated average pairwise relatedness (APR; see methods). (a) APR was weakly correlated with homozygosity-by-loci (HL) but explained only a small proportion of the variance. (b) The correlation between APR and the probability of assignment index (see methods) was much higher. Linear regression lines were calculated using ordinary least squares (N = 224).

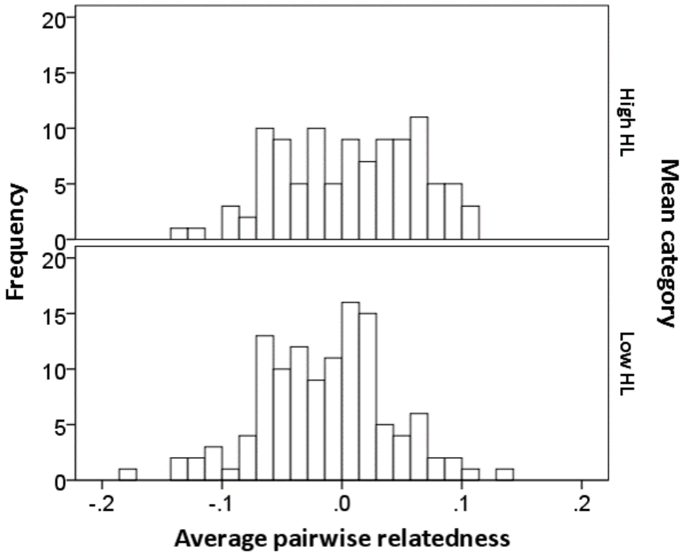

Supplementary Figure S3: The frequency distribution of average pairwise relatedness in two different groups. High HL contains individuals with higher than average (Mean HL = 0.34) homozygosity-by-loci (HL) values and Low HL contains individuals with lower than the average HL.

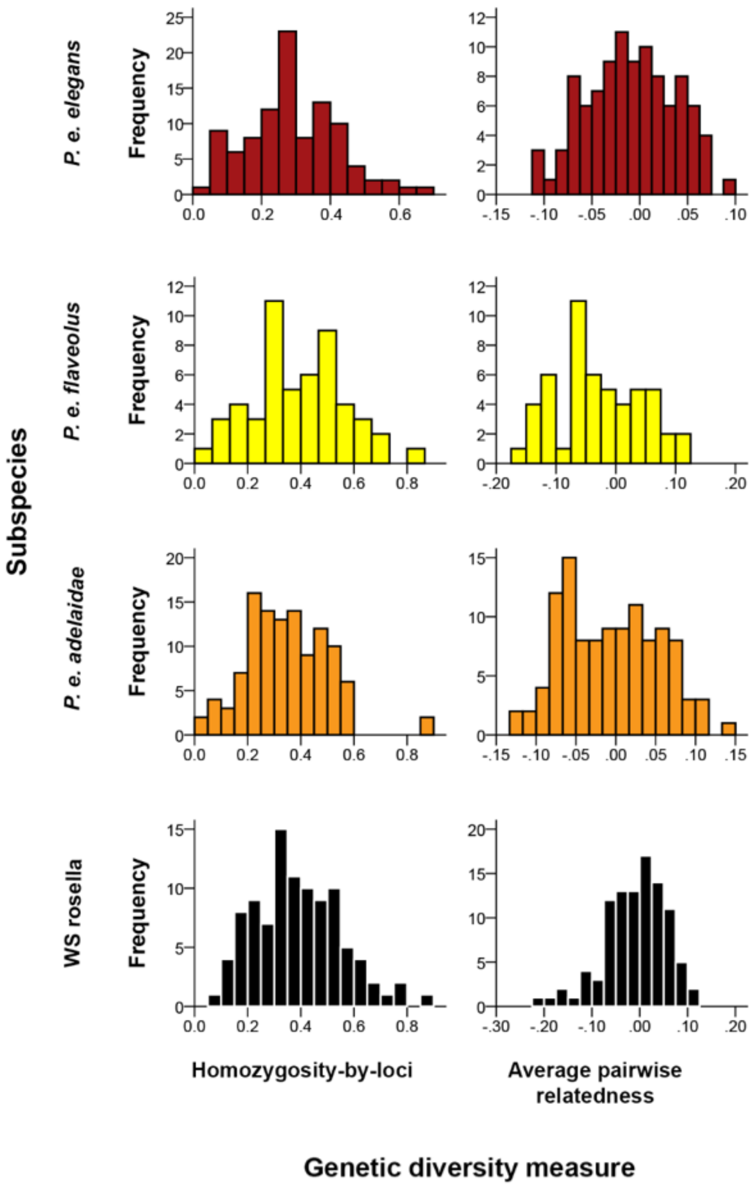

Supplementary Figure S4: Frequency distribution of each individual genetic diversity measure was similar across populations (n = 363 individuals).
